# How the Relativistic Motion Affect Quantum Fisher Information and Bell Non-locality for Multipartite state

**DOI:** 10.1038/srep38456

**Published:** 2017-02-01

**Authors:** Chun Yu Huang, Wenchao Ma, Dong Wang, Liu Ye

**Affiliations:** 1 School of Physics and Material Science, Anhui University, Hefei 230601, P. R. China

## Abstract

In this work, the quantum fisher information (QFI) and Bell non-locality of a multipartite fermionic system are investigated. Unlike the currently existing research of QFI, we focus our attention on the differences between quantum fisher information and Bell non-locality under the relativistic framework. The results show that although the relativistic motion affects the strength of the non-locality, it does not change the physical structure of non-locality. However, unlike the case of non-locality, the relativistic motion not only influence the precision of the QFI *F*_*ϕ*_ but also broke the symmetry of the function *F*_*ϕ*_. The results also show that for a special multipartite system, 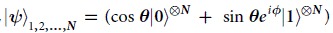, the number of particles of a initial state do not affect the *F*_*θ*_. Furthermore, we also find that *F*_*θ*_ is completely unaffected in non-inertial frame if there are inertial observers. Finally, in view of the decay behavior of QFI and non-locality under the non-inertial frame, we proposed a effective scheme to battle against Unruh effect.

As the two pillars of modern physics, general relativity and quantum mechanics have profoundly influenced our understanding of physical phenomena. General relativity correctly described and predicted the astronomical observations at large spatial scales, and quantum mechanics accurately depicted the experimental results at the microscopic scales. Although these two theories have been generally successful in their respective regimes, a unified theory has not been established. Fortunately, as a burgeoning interdiscipline composed of information theory, quantum field theory, and general relativity, relativistic quantum information not only is considerably important in the long-distance quantum communication, also uncovers a new aspect of the complex relationship between general relativity and quantum mechanics. Therefor, it has attracted extensive attention in the field of quantum communication.

In this context, a great deal of research has been devoted to study the influence of relativistic effects on quantum entanglement^1^,^2^. Unruh or Hawking effect has been a focus of research in recent years^3^,^4^. The corresponding work greatly promote theoretical research and practical application in the field of quantum communication. More specifically, since Peres *et al*.^5^ published their work on quantum entropy and the relativity theory, many people have paid their attentions to the investigation of quantum information in the non-inertial frame^6^,^7^,^8^,^9^,^10^,^11^. For example, adesso *et al*.^12^ investigated the distribution of entanglement sharing of a scalar field in a relativistic setting and proposed a explanation about entanglement redistribution. Moreover, they also shown that the classical correlations is immune to the Unruh effect if one or more observers stays stationary. Wang *et al*.^13^ discussed classical and quantum correlation sharing of a Dirac field in a relativistic setting and shown that unlike the result of scalar field, classical correlation decrease with the increase of acceleration, i.e., classical correlation will be affected due to Unruh effect even if there are inertial observers.

On the other hand, we also noticed that more and more people have paid their attentions to the investigation of QFI in recent years^14^,^15^,^16^,^17^,^18^,^19^,^20^,^21^,^22^. As an important quantitative method for quantum estimation theory, quantum fisher information plays a vital role in quantum information theory and is widely used in quantum information processing^23^,^24^,^25^,^26^,^27^,^28^,^29^,^30^,^31^. Therefore, it will be interesting to discuss QFI in a relativistic framework. Moreover, some people have already attempted to study related issues^32^,^33^,^34^. Recently, Yao *et al*.^34^ investigated the QFI of two-qubit systems for both scalar and Dirac fields when one observer is accelerated and shown that for both cases, the QFI with respect to different state parameters exhibit different properties. Concretely, *F*_*ϕ*_ shows a decay behavior with the increase of the acceleration, while *F*_*θ*_ is completely unaffected by acceleration. As a further step along this line, we here will focus on exploring the performance of QFI and non-locality of Dirac particles in multipartite systems when more than one observer are accelerated. Unlike the previous studies, we will mainly research the difference between QFI and non-locality of a multipartite state in non-inertial frame. To do so, we take the parametrized state as the initial state, characterized by two parameters *θ* and *ϕ*.

## Results

### Physical model and probe state preparation

We assume that Alice, Bob and Charlie share a generically tripartite entangled state at the some initial point in flat Minkowski space-time. Alice has a detector that only detects mode |*n*〉_*A*_, and Bob and Charlie have their detectors sensitive only to modes |*n*〉_*B*_ and |*n*〉_*C*_, respectively. Here, we choose the tripartite entangled initial state as following:


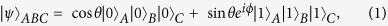


where *θ* and *ϕ* are respectively the weight and phase parameters. Using Eg. (14), one can easily estimate these two parameter and obtain the corresponding quantum fisher information


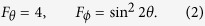


Surprisingly, both of these results are exactly same as values as shown in Ref. [34]. Note that our study was conducted in tripartite system while the former in bipartite case, is this coincidence or intrinsic property? We will present a detailed description in the next section. Furthermore, it is worth pointing out that *F*_*θ*_ is constant which is independent of weight parameter *θ*, the function *F*_*ϕ*_ is symmetric with respect to *θ* = *π*/4.

Now we assume that Alice remain stationary, Bob and charlie move with a uniform acceleration. Since Bob and Charlie undergo uniform acceleration and become uniformly accelerated observers, the state corresponding to modes |*n*〉_*B*_ and |*n*〉_*C*_ must be specified in Rindler coordinates (*τ, ζ*) in order to describe what they see^35^. Therefore, both of these modes will be expanded into rindler regions *I* and *II* respectively. Explicitly, the Minkowski vacuum and single excitation state can be expressed in terms of Rindler modes as follows^36^,^37^


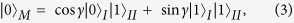



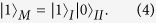


where 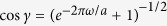, *ω* is the frequency of the Dirac particle, *a* is acceleration, and the acceleration parameter *γ* is in the range 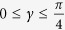 corresponding to 0 ≤ *a* < ∞.

### The Behaviors of quantum fisher information when two observers are accelerated

In the light of the previous discussion, we know that after Bob and Charlie are accelerated, the initial state can be rewrite in terms of Minkowski modes for Alice and Rindler modes for Bob and Charlie:


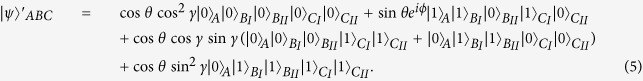


Since Bob and Charlie move with a uniform acceleration, they are causally disconnected from Rindler region *II* and has no access to field modes in this region. Tracing over the inaccessible modes *B*_*II*_ and *C*_*II*_, we obtain the reduced density matrix


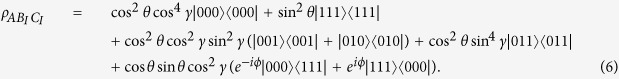


According to the calculation process of QFI, one can easily obtain the all information needed to calculate the QFI. Through calculations, *F*_*θ*_ and *F*_*ϕ*_ are given by


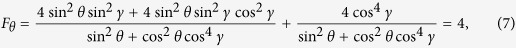



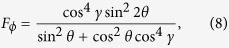


respectively. Obviously, *F*_*θ*_ remains invariant. Interestingly, we note that *F*_*θ*_ is equal to 4 when one observer is accelerated, as shown in the Ref. [34]. Furthermore, we have pointed out in the previous section that the same *F*_*θ*_ have been obtained in the bipartite and tripartite systems. In order to explain this phenomenon, we have studied various quantum states in different situations. Then, we find that for a special quantum states, 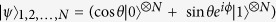, the number of particles of a initial state do not affect the QFI *F*_*θ*_, that is to say, *F*_*θ*_ is independent of the number of particles of the initial state. Moreover, we also find that *F*_*θ*_ is completely unaffected in non-inertial frame if there are inertial observers. This is a remarkable result should be concerned in the field of quantum communication. Because it implies that if we encode the information on the weight parameter, the corresponding accuracy of the parameter estimation will not be affected by non-inertial observer. Nevertheless, this result cannot simply be understood as *F*_*θ*_ is immune to Unruh effect. First, it is established only for special quantum state, not all of quantum state. Second, *F*_*θ*_ is affected due to Unruh effect when all the observations are accelerated. Therefore, various problems in further work may also need to be overcome. The corresponding research of solving these problems is an interesting question that goes beyond the scope of our work.

**Figure 1:**
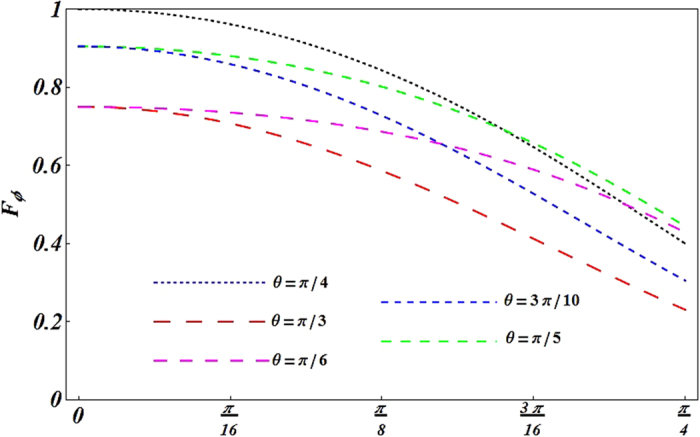
For the state two observers are accelerated, the QFI *Fϕ* as a function of the acceleration parameter *γ* for different *θ*.

**Figure 2:**
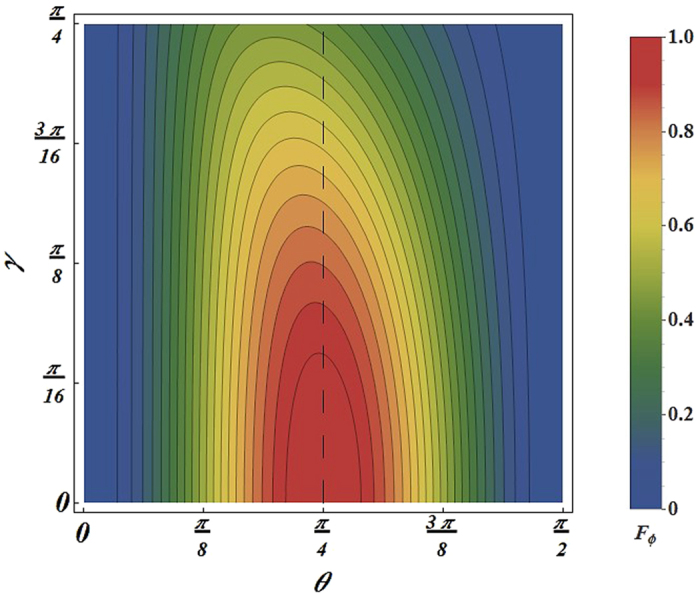
For the state two observers are accelerated, the contour plot of *F*_*ϕ*_ as function of the acceleration parameter *γ* and *Weight* parameter *θ*.

For the *F*_*ϕ*_, one can check that if the acceleration parameter *γ* → 0, i.e., the Unruh effect is nonexistent, *F*_*ϕ*_ is equal to sin^2^ 2*θ*, which is consistent with the case of initial state. The properties of *F*_*ϕ*_ is plotted in Fig. 1 with *ω* = 1. Remarkably, we can gain some interesting results as follows: (i) *F*_*ϕ*_ shows a decay behavior in the non-inertial frame. Furthermore, after a study of multiparticle state 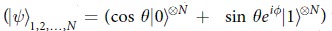 under the non-inertial frame, we find that as the non-inertial observers increase, *F*_*ϕ*_ is affected more significantly and decreases more quickly with the increases of the acceleration *a*. (ii) We find that in the range 0 < *θ* ≤ *π*/4, *F*_*ϕ*_ of initial state increases with the increases of the weight parameter *θ*. Thus, *F*_*ϕ*_ reaches its maximum when 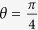. However, when one of observers is accelerated, *F*_*ϕ*_ can’t reaches its maximum when 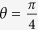. In the limit of infinite acceleration, *F*_*ϕ*_ is monotone decreasing function in the range 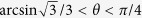. When two observers are accelerated as shown in Eq. (6), the interval expanded to 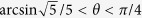. If the number of non-inertial observers continue to increase, the interval will be bigger and bigger. (iii) The symmetry of the function *F*_*ϕ*_ with respect to *θ* = *π*/4 has been broken as we can see from Fig. 2.

### Comparison with Bell Nonlocality

In this work, we choose the Svetlichny inequality (*I*) as the quantification of the tripartite nonlocality. For the initial state in Eq. (1), the *I* value is


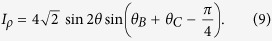


The maximum value is 
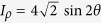
 when 
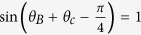
. In order to understand the performance of nonlocality under the non-inertial frame, we also calculate the *I* value of the state in Eq. (6), the *I* value is


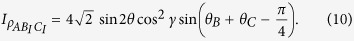


**Figure 3:**
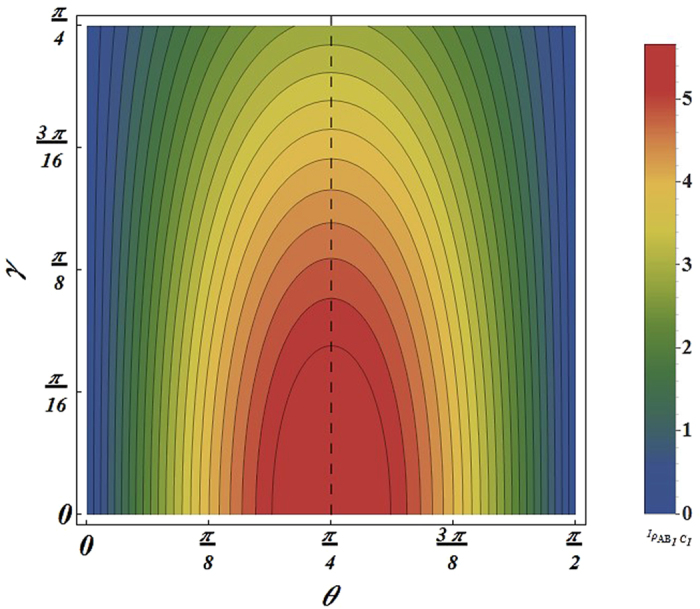
For the state two observers are accelerated, the nonlocality expressed as 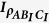 value in term of *Weight* parameter *θ* and acceleration parameter *γ*.

It is clearly that 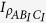 is equal to 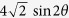 when *γ* = 0, which is exactly the value of the initial state. Moreover, the function 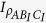 is monotone decreasing with the increase of acceleration, which is similar to the behavior of *F*_*ϕ*_. However, unlike the case of *F*_*ϕ*_ under the non-inertial frame, the symmetry of the function 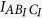 is still not be broken as shown in Fig. 3. This means that although the non-locality shows a decay behavior with the increasing of the acceleration *a*, its physical structure is not broken due to the Unruh effect. Hence, QFI is better than non-locality for reveals intricate and subtle behavior of quantum system.

### Enhancing quantum fisher information and nonlocality by utilizing local filtering operations

In the light of previous discuss, we know that Quantum fisher information and Non-locality show a decay behavior with the increasing of the acceleration *a*. Thus it is of great importance to battle against Unruh effect before using quantum state. Therefore, we propose a scheme to enhance the quantum fisher information and multipartite nonlocality by a single local filtering operation.

Firstly, Bob and Charlie will implement a POVM measurement on their corresponding qubit. After this POVM measurement event, the state which is under the non-inertial frame can be expressed as


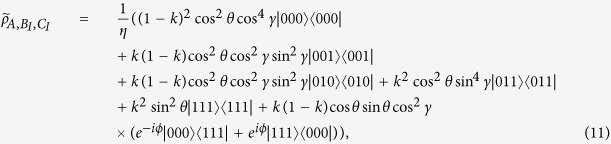


where 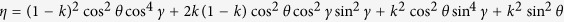. Through calculation, *F*_*ϕ*_ and *I* of output state are given by


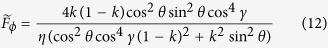



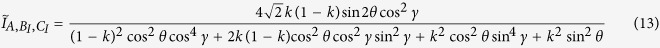


**Figure 4:**
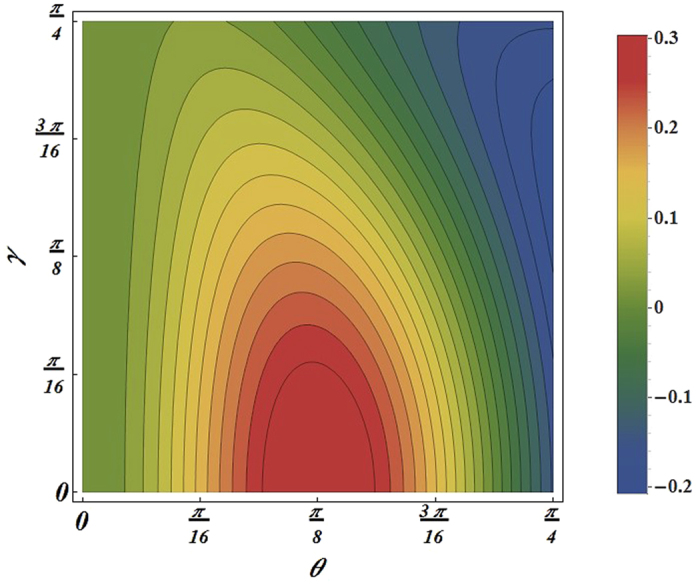
The quantum fisher information difference 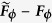 as functions of the Weight parameter *θ* and the acceleration parameter *γ*.

**Figure 5:**
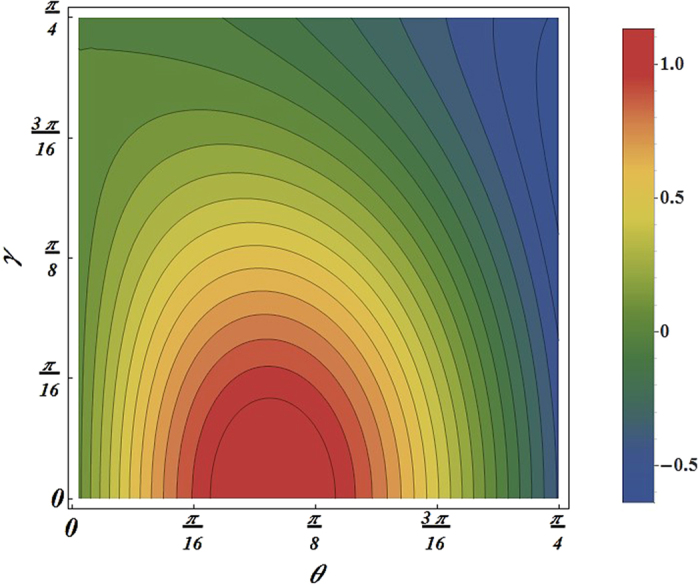
The *I* value difference 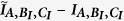 as functions of the Weight parameter *θ* and the acceleration parameter *γ*.

To clearly see the protection effects of the scheme, we plot the quantum fisher information difference 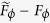 and *I* value difference 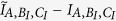 as functions of the Weight parameter *θ* and the acceleration parameter *γ* in Figs 4 and 5, respectively. In particular, we choose the filtering operation parameter *k* = 0.6. From the plot we can see that there are areas where the protection scheme succeed, i.e., 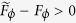 or 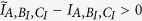. Therefore, the states with the parameters in those area can be protected by the filtering operation. For example, for the input state of 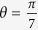, the violation of Svetlichny inequality can be satisfied when the acceleration parameter in the range 0 < *γ* < *π*/10. After the filtering operation, for the output state of 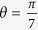, the Svetlichny inequality is violated when the acceleration parameter in the range 0 < *γ* < 2*π*/15. This implies that in the interval *π*/7 < *γ* < 2*π*/15 the violation of Svetlichny inequality is possible for the output state but is impossible for the input state, so our scheme can effectively suppress Unruh effect.

## Discussion

We have investigated how the relativistic motion affect the quantum fisher information and non-locality of the Dirac particles. It has been verified that the QFI with respect to weight parameter *θ* is independent of the acceleration *a* when there are inertial observers. Moreover, we also find that for a special multipartite states, 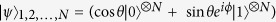, the number of particles of the initial state do not affect the QFI *F*_*θ*_, that is to say, *F*_*θ*_ is independent of the number of particles of the initial state. Both of these results are meaningful in the practical application. Since quantum information processing is inevitably implemented under the non-inertial frame, moreover, the realization of quantum networks ineluctably need multiparticle entangled state, so one can encoded the information on the weight parameter to perform the quantum information task. Nevertheless, these results do not mean that *F*_*θ*_ is immune to Unruh effect. The specific reasons have been given in the previous discussion. On the other hand, although relativistic motion will not affect *F*_*θ*_, but it will greatly influence on *F*_*ϕ*_. It has been shown that *F*_*ϕ*_ shows a decay behavior with the increase of acceleration *a*. Similarily, the non-locality also shows a decay behavior with the increase of the acceleration. In addition, the result also shown that in the range 0 < *θ* ≤ *π*/4, *F*_*ϕ*_ of initial state monotonically increases with the increases of the weight parameter *θ* and reaches its maximum when 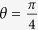. However, when one of observers is accelerated, *F*_*ϕ*_ can not reaches its maximum when 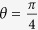. In the limit of infinite acceleration, *F*_*ϕ*_ is a monotone decreasing function in the range 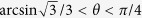. When two observers are accelerated as shown in Eq. (6), the interval expanded to 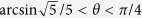. Moreover, for Multipartite states, 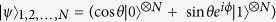, as the number of non-inertial observers increase, the interval will expanded further. Next, we analyzed the properties of nonlocality under the non-inertial frame. Unlike the case of QFI *F*_*ϕ*_, although the non-locality shows a decay behavior with the increasing of the acceleration *a*, its physical structure is not broken due to the Unruh effect. The symmetry of the function *I* has not damaged. But the symmetry of the function *F*_*ϕ*_ with respect to *θ* = *π*/4 has been broken due to the influence of acceleration. Since QFI characterizes the performance of a specific parameters rather than the whole quantum state, so we think that QFI is better than non-locality for reveals intricate and subtle behavior of quantum system. Finally, in view of the decay behavior of QFI and non-locality under the non-inertial frame, we proposed a scheme to battle against Unruh effect. The results shown that our scheme can indeed be useful for combating Unruh effect, and recovering the quantum nonlocality and quantum fisher information.

## Methods

### Quantum Fisher Information

We introduce a useful form of QFI for a special kind of N-dimensional quantum states *ρ*_*λ*_, depending on an unknown parameter *λ*. Based on the spectrum decomposition 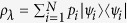, where {|*ψ*_*i*_〉} forms an orthonormalized and complete basis, with *p*_*i*_ being the weight of |*ψ*_*i*_〉, the QFI can be written as^38^


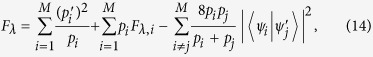


where 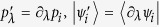, and 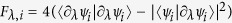. Obviously, QFI can be divided into three parts: the first term is the classical Fisher information for a probability distribution. The second term is a weighted average of quantum Fisher information over all pure state. The last term is from the mixture of pure states and thus decreases the total QFI.

### The Svetlichny inequality

According to Ref. 39, the Svetlichny inequality is defined as follows


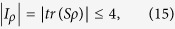


in terms of the Svetlichny operator^40^


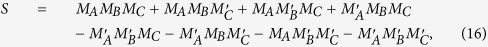


where the measurement operators *M*_*i*_ and 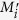 correspond to the measurements on each of the qubit (*i* = *A, B, C*), the primed and unprimed terms denote two different measurement directions. For a tripartite quantum state, the svetlichny inequality is violatied whenever |*I*_*ρ*_| > 4, and in quantum mechanics the svetlichny inequality is violated up to 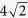. The measurement operators on the second qubit differ by *θ*_*i*_ from those performed on the first one:


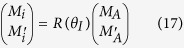


with


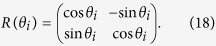


There are two such rotation angles *θ*_*B*_ and *θ*_*C*_. We define *M*_*A*_ = *σ*_y_ and 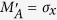, and the corresponding measurement operators are given by


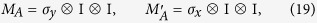



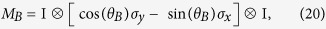



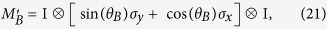



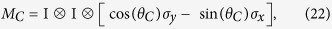



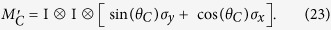


### The filtering operation

This operation has a greater experimental feasibility than some other common measurements, and it is a Non-Trace-Preserving map (NTPM) which is known to be capable of increasing entanglement with some probability^41^. The filtering operation can be written in a computational basis as


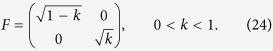


When filtering operation of Eq. (24) is applied to the all qubits, the final states 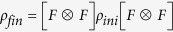.

## Additional Information

**How to cite this article**: Huang, C. Y. *et al*. How the Relativistic Motion Affect Quantum Fisher Information and Bell Non-locality for Multipartite state. *Sci. Rep.* **7**, 38456; doi: 10.1038/srep38456 (2017).

**Publisher's note:** Springer Nature remains neutral with regard to jurisdictional claims in published maps and institutional affiliations.
